# 多孔有机笼的制备及其用作毛细管电色谱手性固定相

**DOI:** 10.3724/SP.J.1123.2023.05008

**Published:** 2023-10-08

**Authors:** Kangni ZHENG, Gaizhao QIN, Xuefei JIANG, Junhui ZHANG, Liming YUAN

**Affiliations:** 云南师范大学化学化工学院, 云南 昆明 650500; School of Chemistry and Chemical Engineering, Yunnan Normal University, Kunming 650500, China

**Keywords:** 毛细管电色谱, 多孔有机笼, 手性分离, 手性药物, capillary electrochromatography (CEC), porous organic cage (POC), chiral separation, chiral drugs

## Abstract

多孔有机笼(POCs)是具有一类内在的、客体可及的空腔的离散分子,是一类独特的微孔材料。本文根据席夫碱反应原理,用(1*R*,2*R*)-二氨基环己烷和3,3',5,5'-四醛基-4,4'-联苯二酚缩合成一种孔径均匀、高比表面积、热化学稳定性良好的棱柱形手性多孔有机笼(POCs)。采用核磁共振氢谱仪、红外光谱、热重分析和扫描电子显微镜对该材料进行表征。将该材料溶解在二氯甲烷中,用动态涂覆法将溶液均匀地涂覆在石英毛细管内壁上,制成毛细管电色谱柱。结果表明,该手性电色谱柱不仅能拆分氧氟沙星、特罗格尔碱、2-氨基-1-丁醇和1-苯基-1-戊醇4种手性药物,还能拆分*o*,*m*,*p*-甲苯胺和*o*,*m*,*p*-氯苯胺2种位置异构体,说明该手性柱具有良好的手性分离能力。通过研究氧氟沙星、特罗格尔碱、2-氨基-1-丁醇和1-苯基-1-戊醇的最佳拆分条件,得出电压、缓冲溶液浓度和pH值对组分分离度有显著影响,其中,氧氟沙星、特罗格尔碱、2-氨基-1-丁醇的最优分离电压均为15 kV, 1-苯基-1-戊醇的最佳分离电压为17 kV;氧氟沙星、特罗格尔碱、2-氨基-1-丁醇、1-苯基-1-戊醇的最佳缓冲液浓度均为0.100 mol/L, pH为7.5。在最佳的分离条件下,该电色谱柱拆分氧氟沙星、特罗格尔碱、2-氨基-1-丁醇和1-苯基-1-戊醇的分离度分别为1.80、3.33、1.69、1.18,都获得了较好的分离效果。可见,POCs是一种良好的手性毛细管电色谱固定相,有一定的手性拆分能力,在色谱分离中具有良好的应用前景。

## Abstract (en)

Porous organic cages (POCs) are a unique type of microporous materials composed of discrete molecules with internal cavities that are accessible to various compounds. In this study, a prismatic chiral POC with good thermochemical stability was synthesized by condensing (1*R*,2*R*)-diaminocyclohexane and 3,3',5,5'-teturonic-4,4'-biphenediol via the Schiff base reaction and characterized by proton nuclear magnetic resonance spectroscopy, infrared (IR) spectroscopy, thermogravimetric analysis (TGA), and scanning electron microscopy. The IR spectrum of the POC revealed a strong characteristic absorption peak at 1635 cm^-1^, indicating that it formed imine bonds (C=N). The absorption peak at 3425 cm^-1^ was attributed to the stretching vibrations of -OH, the absorption peaks at 2925 and 2858 cm^-1^ were attributed to the stretching vibrations of N=C-H and C-H, and the absorption peaks at 1446 and 1383 cm^-1^ were attributed to the stretching vibrations of C=C-H and C=C in the benzene ring. High-resolution mass spectral analysis of the POC showed a molecular ion peak at *m/z* 1363.7228, indicating its successful synthesis. TGA was performed from 25 to 800 ℃ at a rate of 10 ℃/min, and the results of this analysis showed that the POC was stable up to approximately 300 ℃. The POC was dissolved in dichloromethane and uniformly coated on the inner wall of a quartz capillary via the dynamic coating method to prepare a capillary electrochromatographic column. The experimental results revealed that the chiral electrochromatographic column could not only resolve ofloxacin, Troger’s base, 2-amino-1-butanol, and 1-phenyl-1-amyl alcohol but also separate the isomers of *o*-, *m*-, and *p*-toluidine and *o*-, *m*-, and *p*-chloroaniline, indicating its good chiral separation ability. Investigation of the optimal separation conditions for ofloxacin, Troger’s base, 2-amino-1-butanol, and 1-phenyl-1-amyl alcohol revealed that the voltage, buffer solution concentration, and pH significantly affected their separation degree. In particular, the optimal separation voltage for ofloxacin, Troger’s base, and 2-amino-1-butanol was 15 kV, while that for 1-phenyl-1-amyl alcohol was 17 kV. The optimal buffer concentration and pH for ofloxacin, Troger’s base, 2-amino-1-butanol, and 1-phenyl-1-amyl alcohol were 0.100 mol/L and 7.5. Under optimal chromatographic conditions, the resolution values for ofloxacin, Troger’s base, 2-amino-1-butanol, and 1-phenyl-1-pentanol were 1.80, 3.33, 1.69, and 1.18, respectively. The results collectively demonstrate that the prepared POC may serve as a good chiral stationary phase for capillary electrochromatography with a certain chiral resolution ability and has good application prospects in chromatographic separation.

手性(chirality)一词源于希腊语中的“*cheir*”,它类似于人左右手的关系,即两者互为镜像^[1]^。手性在自然界和生命体中无处不在,如自然界中的龙卷风,生命体中的蛋白质、氨基酸、多糖等都是手性物质。在药理性质方面外消旋体的*R*构型和*S*构型往往有着不同甚至相反的作用,例如甲状腺素钠的右旋体可以作为降血脂药物应用,但其左旋体对心脏具有严重的副作用,又如三唑类杀菌剂(2*R*, 3*R*)构型有高杀菌作用和低等植物生长控制作用,而其(2*S*, 3*S*)构型则有着相反的作用^[1]^。可见对外消旋体的拆分具有极为重要的意义。外消旋体的拆分方法有很多,如色谱法、化学拆分法等,其中,毛细管电色谱(CEC)技术已经逐渐发展成为化学、环保、医药等领域广泛应用且有效的色谱分离方法^[2]^。CEC技术是通过在毛细管柱内涂覆或键合手性固定相,以电渗流为驱动力,根据对映体在手性固定相和流动相之间分配系数及电泳淌度的不同实现分离^[3]^。CEC的手性固定相种类众多,其中多孔有机笼(POCs)就是其中的一种,POCs因其固有的和可接近的空腔,成为一类独特的孔状材料^[4]^。

POCs有着孔径均匀、表面积高、热化学稳定性好的性质,这使其成为各种功能应用的最佳候选者,包括分子气体分离和手性药物分离^[5]^。在固体中呈现多孔的状态,且空腔必须通过一维、二维或三维的孔隙网络来连接,如果没有这种连通性,固有的空腔是孤立的,客体分子就无法进入^[6]^。有机笼在添加和去除客体时也必须保持形状,如溶剂的孔隙网络结构会因其内在腔体的崩溃而受到破坏,笼内的内在孔隙度也可能因笼间的外部空隙而增大^[7⇓-9]^。与金属有机框架、沸石、多孔聚合物和碳分子筛等多种多孔材料相比,POCs是通过共价键合自组装形成的具有三维连通性和均匀孔径的晶体多孔材料^[10⇓-12]^。2008年,Gawronski课题组^[13]^通过缩合反应,合成了一个连接亚胺的[4⇓+^6^]四面体笼,但他们并没有研究笼的晶体结构。Yuan课题组^[14]^在2015年报道了多孔有机分子笼CC3-R作为毛细管气相色谱手性固定相对手性物质具有很好的分离效果;Kewley等^[15]^报道了POCs在气相色谱和电色谱中的应用,证实了POCs是一种不错的手性材料;2021年,Yuan课题组的唐明华^[16]^制备了两种手性POCs,由2-羟基-1,3,5-均苯三甲醛分别与(1*R*,2*R*)-二氨基环己烷和(1*R*,2*R*)-二苯乙二胺合成,两种手性POCs的合成反应都是席夫碱反应。即用伯胺与醛或者酮发生席夫碱反应获得有机分子胺笼^[17]^。之后把它们用作CEC的手性固定相,拆分了多种手性药物和位置异构体,达到了预期的效果。

文章利用席夫碱反应原理,通过溶剂热合成法将(1*R*,2*R*)-二氨基环己烷和3,3',5,5'-四醛基-4,4'-联苯二酚缩合成棱柱形手性POCs。该POCs具有良好的溶解性,可溶于大多数有机溶剂,适合作为CEC的手性固定相。将POCs溶解在二氯甲烷中,用动态涂覆法将溶液均匀地涂覆在石英毛细管内壁上,制成CEC柱。实验结果表明该手性柱拆分了4种手性药物和2种位置异构体,说明该手性柱具有一定的手性分离能力。

## 1 实验部分

### 1.1 仪器与试剂

CL 1020高效毛细管电泳仪(北京华阳利民仪器有限公司); Bruker DXR 500 MHz核磁共振波谱仪(瑞士布鲁克公司); D/Max 2000粉末衍射仪(日本Rigaku公司); Vario EL Ⅲ有机化学元素分析仪(北京来亨科贸有限责任公司); SDT-650热重分析仪(美国TA仪器); XL 30 ESEM-TMP扫描电子显微镜(荷兰飞利浦公司);纯水器(英国ELGA公司)。

氢氧化钾、二氯甲烷、乙醇、甲醇、盐酸、二甲基亚砜、联苯二酚、磷酸购于天津风船化学试剂有限公司;(1*R*,2*R*)-环己二胺、三氟乙酸、六亚甲基四胺、*o*,*m*,*p*-氯苯胺、*o*,*m*,*p*-甲苯胺购于上海试剂有限公司;氧氟沙星、特罗格尔碱、2-氨基-1-丁醇、1-苯基-1-戊醇购于比利时Acros Organics公司。实验所用试剂纯度除磷酸为85%与(1*R*,2*R*)-环己二胺为98%外,其余均为99%。

### 1.2 配体3,3',5,5'-四醛基-4,4'-联苯二酚的合成

根据文献[18]合成3,3',5,5'-四醛基-4,4'-联苯二酚:首先称取4,4'-联苯二酚6.85 g (16.11 mmol)和已在真空下干燥2.5 h的六亚甲基四胺51.2 g (182.6 mmol),置于500 mL圆底烧瓶中,加入三氟乙酸(TFA) 120 mL,在油浴锅中反应7 d,温度为105 ℃。反应结束后冷却至室温,再加入200 mL浓度为4 mol/L的盐酸,反应2.5 h,再冷却12 h后过滤,用超纯水、无水乙醇、环己烷等有机溶剂依次洗涤。最后真空干燥,得到2.9 g的黄色固体。为了得到更纯的物质,将黄色固体进行两次重结晶,得到纯净的3,3',5,5'-四醛基-4,4'-联苯二酚。化学反应结构式如图1所示:

**图 1:**
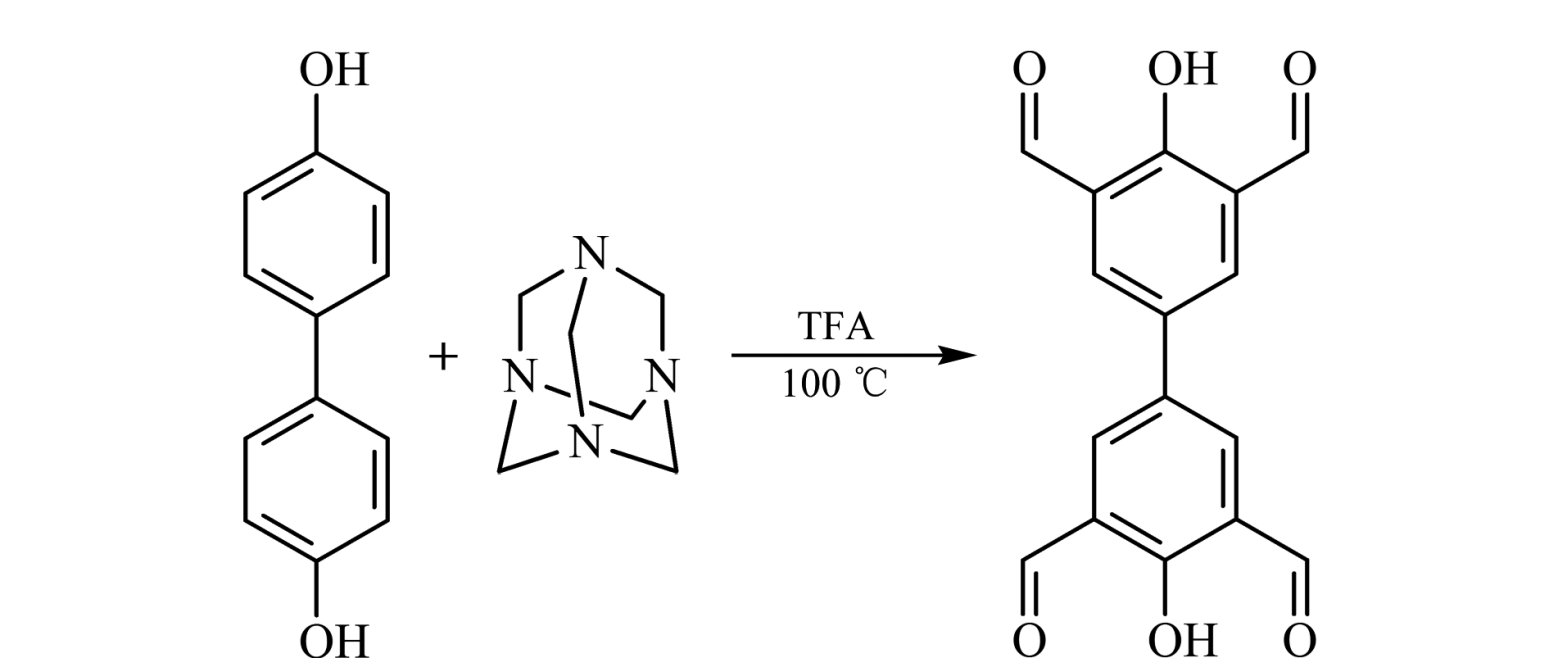
3,3',5,5'-四醛基-4,4'-联苯二酚的合成

### 1.3 POCs的合成

根据文献[18]报道方法合成了POCs。首先,称取上述步骤合成的配体3,3',5,5'-四醛基-4,4'-联苯二酚0.6 g (0.50 mmol)溶于100 mL的*N*,*N*-二甲基甲酰胺(DMF)中,并将2.62 g (1.5 mmol)的(1*R*,2*R*)-二氨基环己烷充分溶解在60 mL甲醇中,分别进行超声,待两种固体溶解后,一起倒入250 mL的圆底烧瓶中,在无水无氧条件下,加入5 mL盐酸,回流过夜,过滤后滤液转移至烧杯中,用滤纸盖住,4~5天后,有橙色晶体析出,然后用环己烷、无水乙醇和高纯水洗涤多次,产物在70 ℃的真空干燥箱中进行干燥。合成路线见图2。

**图 2:**
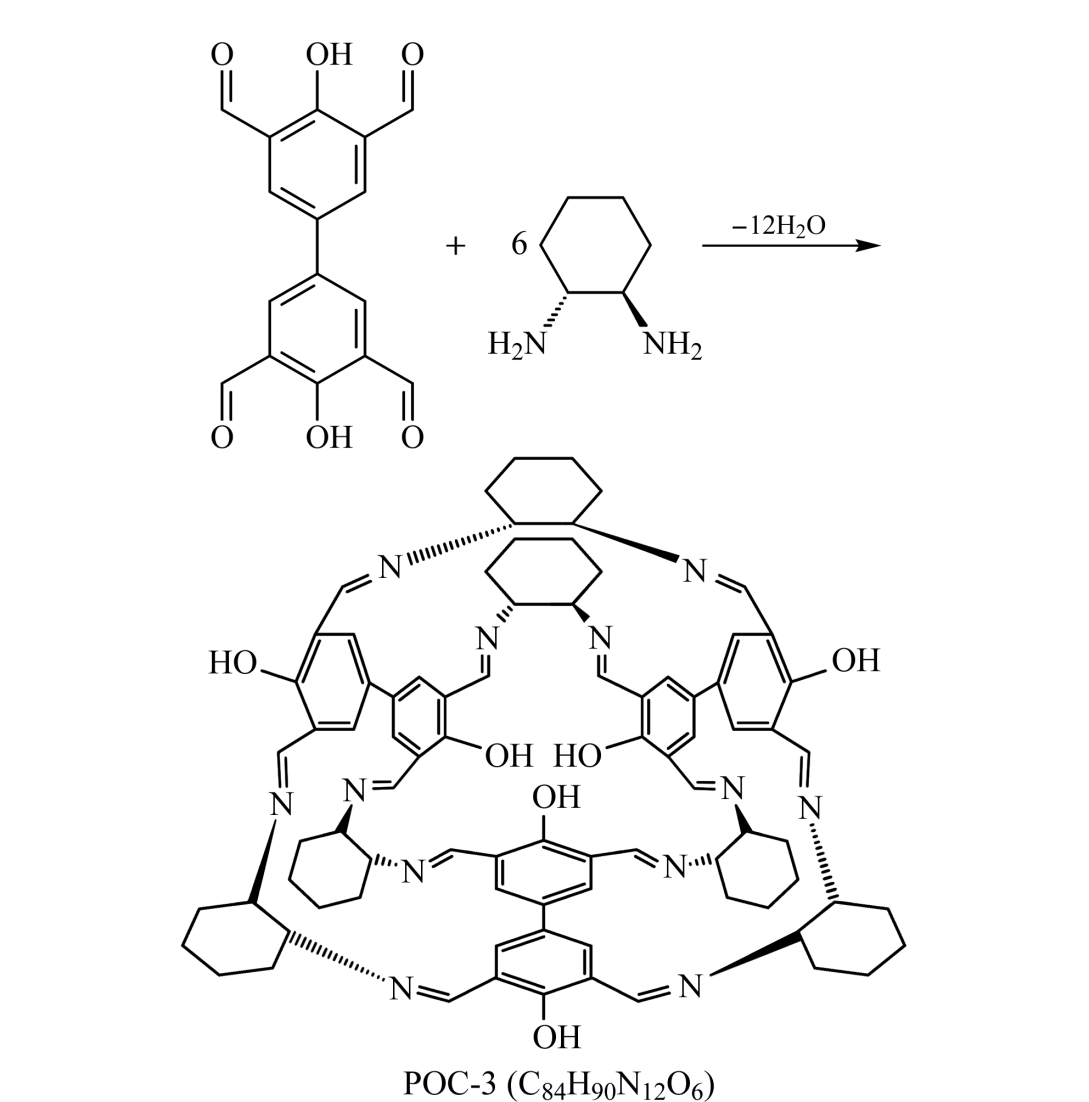
POCs的合成路径

### 1.4 POCs开管毛细管色谱柱的制备

参照文献[19]制备POCs开管毛细管色谱柱,处理步骤如下:先截取大约3 m (375 μm o.d.×75 μm i. d.)长的石英毛细管,然后对其进行粗糙化,具体步骤为依次用1 mol/L NaOH、蒸馏水和0.1 mol/L HCl冲洗,之后将石英毛细管柱烘干,处理温度为120 ℃,烘干时间为40 min,最后将毛细管柱烘干,温度为150 ℃。

称取30 mg的POCs晶体,溶解于10 mL二氯甲烷溶液中,质量浓度为3 mg/mL,超声20 min,充分溶解后,过滤掉不溶固体,接着将上述滤液用高纯氮气缓慢的吹入粗糙化后的毛细管柱中。待充满整个毛细管柱后,再把氮气的气流调小,持续吹3 h,直至溶液吹出并继续对其进行老化。

### 1.5 CEC的实验条件

截取一段手性POCs开管毛细管色谱柱(60 cm×75 μm),在毛细管柱离末端8 cm处进行开窗操作,有效分离长度为52 cm。进行测样之前,色谱柱需用高纯水、磷酸缓冲溶液依次冲洗,直至仪器基线稳定,进样时间为5~10 s。

拆分氧氟沙星、特罗格尔碱、2-氨基-1-丁醇的最优分离电压均为15 kV,而1-苯基-1-戊醇的最优分离电压为17 kV;氧氟沙星、特罗格尔碱、2-氨基-1-丁醇、1-苯基-1-戊醇的适宜Tris-H_3_PO_4_缓冲液浓度均为0.100 mol/L; 4种手性样品拆分时的pH值均为7.5。

## 2 结果与讨论

### 2.1 3,3',5,5'-四醛基-4,4'-联苯二酚的表征

将配体溶解于0.5 mL的氘代二甲亚砜(DMSO-d6)进行核磁共振分析,可以得到配体共有两种不同环境的氢,氢面积之比为1∶1,与参考文献[18]基本一致,表明配体3,3',5,5'-四醛基-4,4'-联苯二酚已成功合成。其中核磁数据如下:^1^H NMR (500 MHz, DMSO): *δ*=8.48 (s, 4H), 10.36 (s, 4H)。

### 2.2 POCs的表征

#### 2.2.1 核磁共振氢谱分析

采用核磁共振氢谱仪对合成的POCs进行了表征,具体数据如下:^1^H NMR (500 MHz, CDCl_3_): *δ*=14.12 (s, 1H), 8.53 (s, 1H), 8.47 (s, 1H), 8.13 (s, 1H), 7.59 (s, 1H), 3.47 (s, 2H, CHN), 3.21 (s, 1H, CHN), 2.05~1.47 (s, 8H)。所得数据与文献[18]报道的相同。

#### 2.2.2 碳谱分析

采用碳谱仪对合成的POCs进行了碳谱表征,具体数据如下:^13^C NMR (125 MHz, CDCl_3_): *δ*=165.11, 161.56, 157.79, 129.07, 126.53, 124.88, 123.82, 118.51, 72.50, 32.43, 31.74, 29.31, 24.22。所得数据与文献[18]报道的相同。

#### 2.2.3 红外光谱分析

采用红外光谱仪对合成的POCs进行表征,如图3所示,强亚胺键在1635 cm^-1^处的特征吸收峰证明了POCs形成了C=N键,在3425 cm^-1^处存在-OH吸收峰伸缩振动,在2925 cm^-1^和2858 cm^-1^处的吸收峰分别为N=C-H和C-H拉伸带,且苯环吸收峰在约1446 cm^-1^和1383 cm^-1^处由C=C-H和C=C拉伸振动产生。

**图 3:**
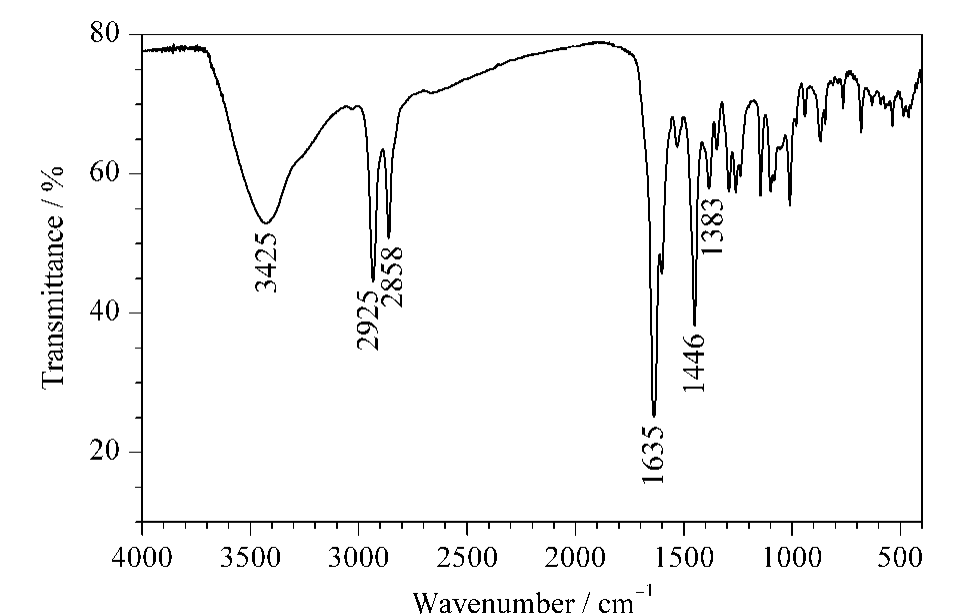
POCs的红外光谱图

通过上述核磁共振氢谱、碳谱与红外光谱的表征及对相关数据的分析,证明实验已经成功合成出了POCs。

#### 2.2.4 质谱分析

POCs分子式为C_84_H_90_N_12_O_6_,理论上:C 74.02%、H 6.59%、N 12.43%。通过元素分析后得到:C 74.12%、H 6.52%、N 12.37%。文章采用高分辨率质谱对POCs进行分析,得到分子离子峰*m/z*=1363.7228(见图4),可推算出其相对分子质量约为1362,符合氮规则,进一步证明已合成出POCs。

**图 4:**
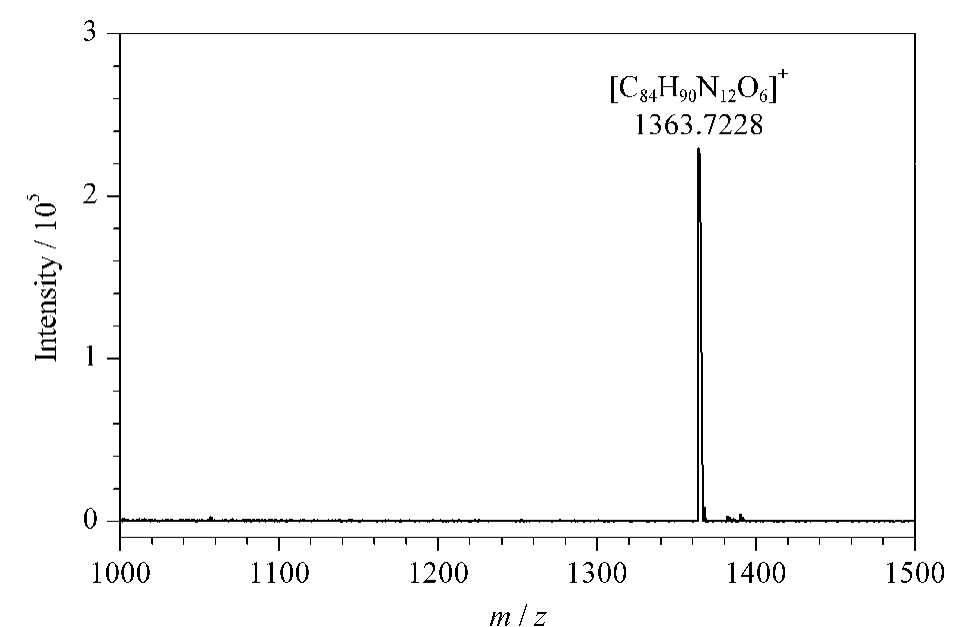
POCs的质谱图

### 2.3 热重曲线分析

为证明POCs有高的化学热稳定性,对POCs进行了热重测试。将POCs放在热重分析仪中,从25 ℃上升到800 ℃(10 ℃/min)。从图5可以观察到,其在300 ℃左右能稳定存在,表明POCs具有良好的化学热稳定性,符合在CEC中的测试条件,可进一步探究其他实验。

**图 5:**
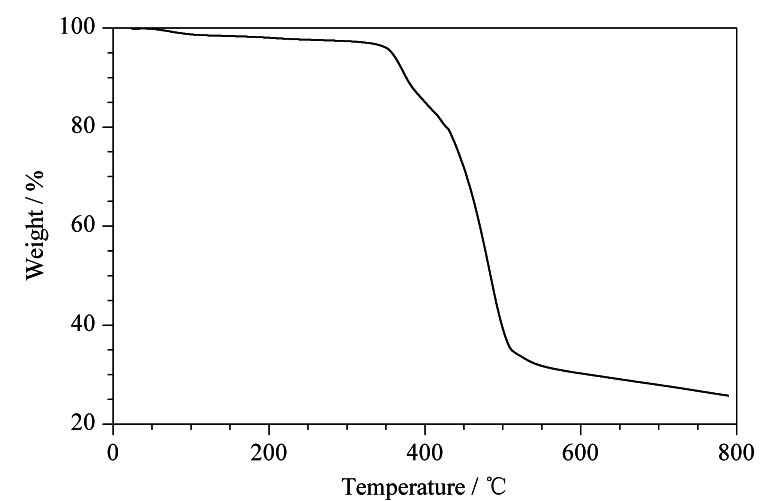
POCs的热重曲线图

### 2.4 POCs的氮气吸附测试

由氮气吸附脱附实验得出,合成的多孔POCs材料比表面积为689.39 m^2^/g,孔径大小为3.91 nm,孔体积为0.41 cm^3^/g,如图6所示。

**图 6:**
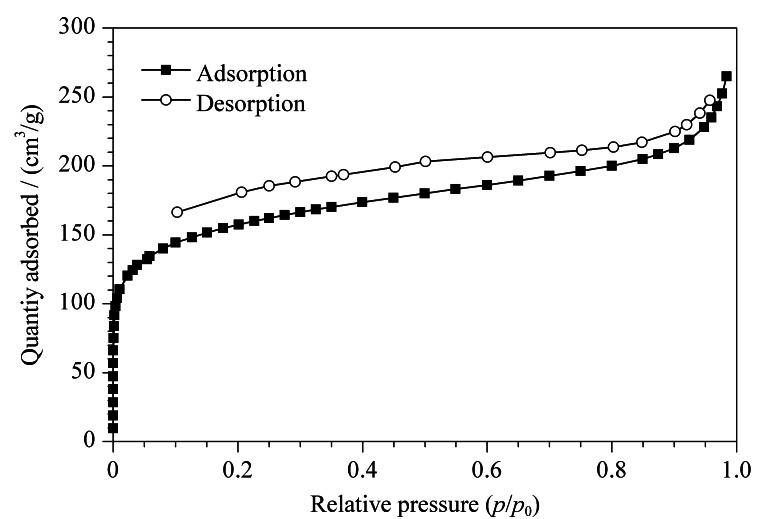
POCs的N_2_吸附脱附等温线

### 2.5 POCs开管毛细管电色谱柱电镜表征

探究POCs开管毛细管电色谱柱是否具有良好的分离性能,首先需要对涂覆好的POCs毛细管色谱柱进行扫描电镜分析表征。图7a、b为空的毛细管柱表征,可观察到其内壁光滑,图7c、d为涂覆柱表征,可以观察到毛细管内壁涂有一层POCs材料。

**图 7:**
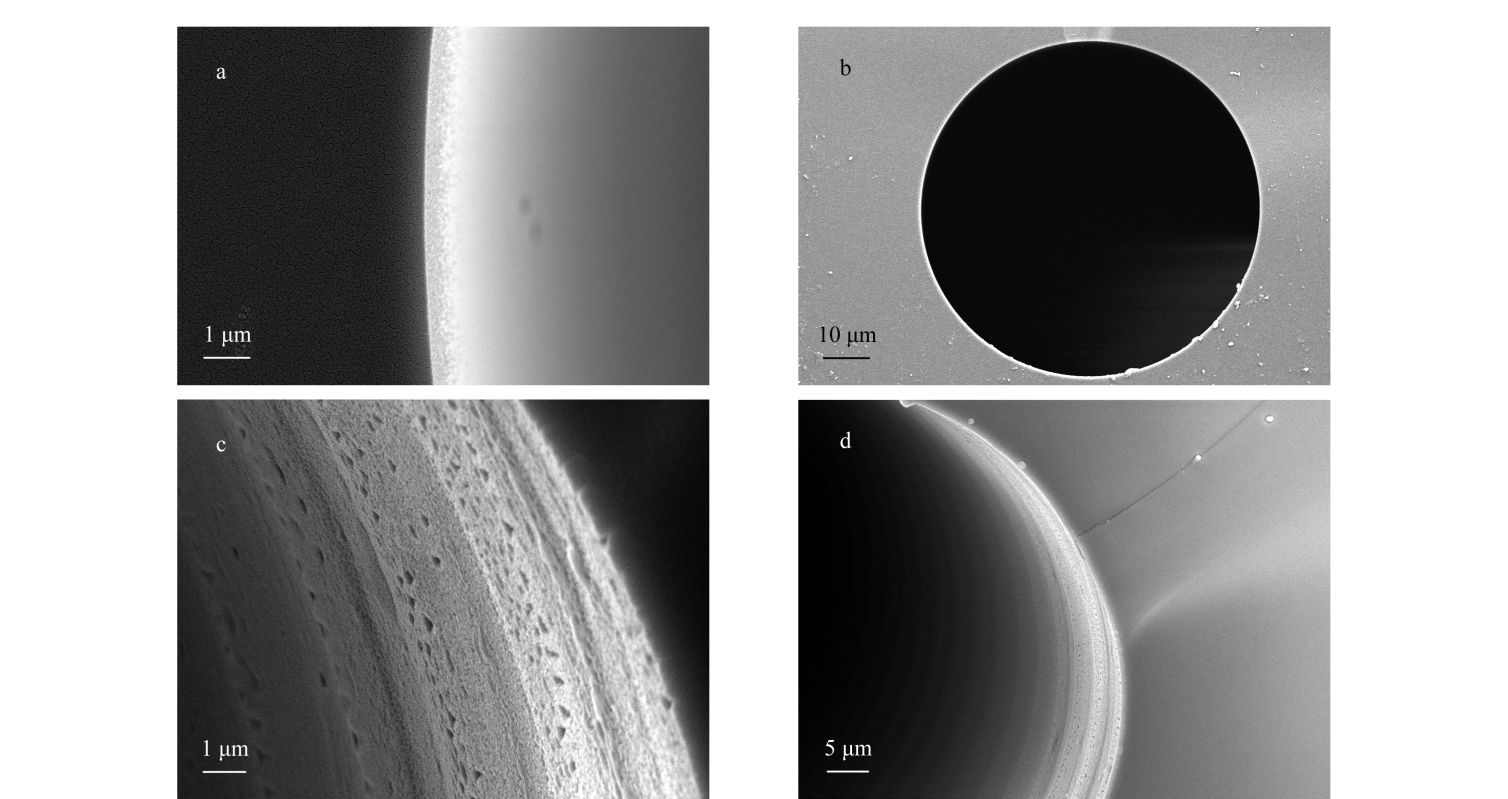
毛细管内壁的扫描电镜图

### 2.6 POCs毛细管电色谱柱拆分手性化合物的情况

POCs具有特别的笼分子和四面体结构,且是一种手性多孔材料。手性药物与有机笼分子之间存在*π-π*键、偶极-偶极作用、立体构型匹配作用和氢键等作用,在进行CEC分离分析时,手性药物进入毛细管内与内壁中涂覆的POCs材料充分接触,在诸多作用下,手性药物得到了分离。然而,手性药物分离的过程十分复杂,很难完全阐明手性分离的机理。为了探究该色谱柱对手性化合物的拆分能力,选用氧氟沙星、特罗格尔碱、2-氨基-1-丁醇与1-苯基-1-戊醇为样品,改变实验中的分离电压、缓冲溶液的浓度、缓冲溶液的pH值,从而得到拆分4种外消旋体的最佳实验条件。

#### 2.6.1 分离电压对手性药物拆分的影响

影响手性药物拆分效果的因素有很多,分离电压就是其中的一种。分离电压的大小不仅影响着手性药物的分离度,且对出峰时间也有一定的影响。为探究分离电压对分离度的影响,选用浓度为0.100 mol/L、pH=7.5的Tris-H_3_PO_4_缓冲溶液分别对氧氟沙星、特罗格尔碱、2-氨基-1-丁醇和1-苯基-1-戊醇4种手性药物进行实验探究,从表1数据可以看出,分离电压为15 kV时,氧氟沙星、特罗格尔碱和2-氨基-1-丁醇能达到最大分离度,1-苯基-1-戊醇在电压为17 kV的条件下达到最大分离度。

**表 1:** 不同分离电压下4种外消旋体的拆分结果

Sample	U/ kV	t_R1_/ min	t_R2_/ min	N_1_/ (plates/m)	N_2_/ (plates/m)	α	R_s_
Ofloxacin	13	31.78	31.05	46713	8806	1.05	0.56
	15	20.74	21.61	42074	86352	1.06	1.80
	17	20.12	20.45	11081	9864	1.04	0.29
Troger’s	13	29.68	32.52	43227	29716	1.05	3.09
base	15	17.86	19.78	52075	23254	1.10	3.33
	17	17.00	18.27	32050	8162	1.07	1.54
2-Amino-	13	16.75	17.30	87567	45771	1.05	1.45
1-butanol	15	13.03	13.42	103876	96842	1.04	1.69
	17	12.45	13.02	35389	42168	1.04	1.60
1-Phenyl-	15	18.64	18.93	102065	39015	1.05	0.69
1-pentanol	17	16.82	17.31	113126	29957	1.05	1.18
	19	10.41	10.76	38061	10562	1.05	0.79

U: separation voltage; N: number of theoretical plates; α: separation factor; R_s_: resolution.

#### 2.6.2 缓冲溶液浓度对手性药物拆分的影响

在最优分离电压条件下,采用pH值为7.5但浓度不同的Tris-H_3_PO_4_缓冲溶液,4种外消旋体的色谱拆分情况如表2所示。实验得出,当缓冲溶液浓度升高到一定程度时,可以增大手性样品的分离度,一旦超过适当的浓度范围,分离度不但不会增大,反而会减小。由表2得出,氧氟沙星、特罗格尔碱、2-氨基-1-丁醇和1-苯基-1-戊醇在POCs手性色谱柱的分离中,最优缓冲溶液浓度为0.100 mol/L。另外,缓冲液浓度影响着样品的出峰时间,样品的出峰时间随着浓度的增大而增加,溶液离子强度的变化对溶液黏度与Zeta电势也有一定的影响。

**表 2:** 不同浓度缓冲溶液下4种外消旋体的拆分结果

Sample	c/ (mmol/ L)	t_R1_/ min	t_R2_/ min	N_1_/ (plates/ m)	N_2_/ (plates/ m)	α	R_s_
Ofloxacin	50	19.62	-	8370	-	-	-
	100	20.74	21.61	42074	86352	1.06	1.80
	150	24.90	25.53	12740	30143	1.04	0.62
Troger’s	50	15.00	-	32400	-	-	-
base	100	17.86	19.78	52075	23254	1.10	3.33
	150	23.36	25.14	74495	25023	1.07	2.70
2-Amino-	50	11.96	-	217670	-	-	-
1-butanol	100	13.03	13.42	103876	96842	1.04	1.69
	150	14.67	-	495646	-	-	-
1-Phenyl-	50	10.48	10.69	49293	19822	1.05	0.62
1-pentanol	100	16.82	17.31	113126	29957	1.05	1.18
	150	17.99	18.47	71208	33356	1.05	1.03

#### 2.6.3 pH值对手性药物拆分的影响

上述实验得到手性药物的最佳分离电压和缓冲液浓度,继续对手性药物的最佳pH值进行优化(见表3),缓冲液pH值从6.5升至7.5时,氧氟沙星、特罗格尔碱、2-氨基-1-丁醇和1-苯基-1-戊醇的分离度升高,但当pH值升高到8.5时,氧氟沙星、1-苯基-1-戊醇的分离度分别减少至0.62、1.06,特罗格尔碱、2-氨基-1-丁醇没能得到拆分。pH值过低会影响色谱固定相的活性,但pH值过高则会增大电渗流,缩短手性样品的保留时间,从而影响拆分效果。因此最优pH值为7.5。

**表 3:** 不同pH值缓冲溶液下4种外消旋体的拆分结果

Sample	pH	t_R1_/ min	t_R2_/ min	N_1_/ (plates/m)	N_2_/ (plates/m)	α	R_s_
Ofloxacin	6.5	27.17	28.42	8535	37338	1.05	1.02
	7.5	20.74	21.61	42074	86352	1.06	1.80
	8.5	17.82	18.71	4066	6320	1.04	0.62
Troger’s	6.5	25.31	27.53	38812	20346	1.05	2.49
base	7.5	17.86	19.78	52075	23254	1.10	3.33
	8.5	14.70	-	16817	-	-	-
2-Amino-	6.5	13.85	14.42	40628	55390	1.05	1.60
1-butanol	7.5	13.03	13.42	103876	96842	1.04	1.69
	8.5	12.15	-	84990	-	-	-
1-Phenyl-	6.5	17.45	17.87	89529	45975	1.05	1.06
1-pentanol	7.5	16.82	17.31	113126	29957	1.05	1.18
	8.5	10.43	10.58	62625	14623	1.05	0.43

经过上述3个实验的研究探讨,得到4种手性物质在最佳拆分条件下的电色谱图如图8所示,氧氟沙星、特罗格尔碱、2-氨基-1-丁醇和1-苯基-1-戊醇的手性分离度分别是1.80、3.33、1.69、1.18,都达到了较好的分离,表明POCs手性色谱柱对外消旋体具有良好的手性识别能力和分离效果。

**图 8:**
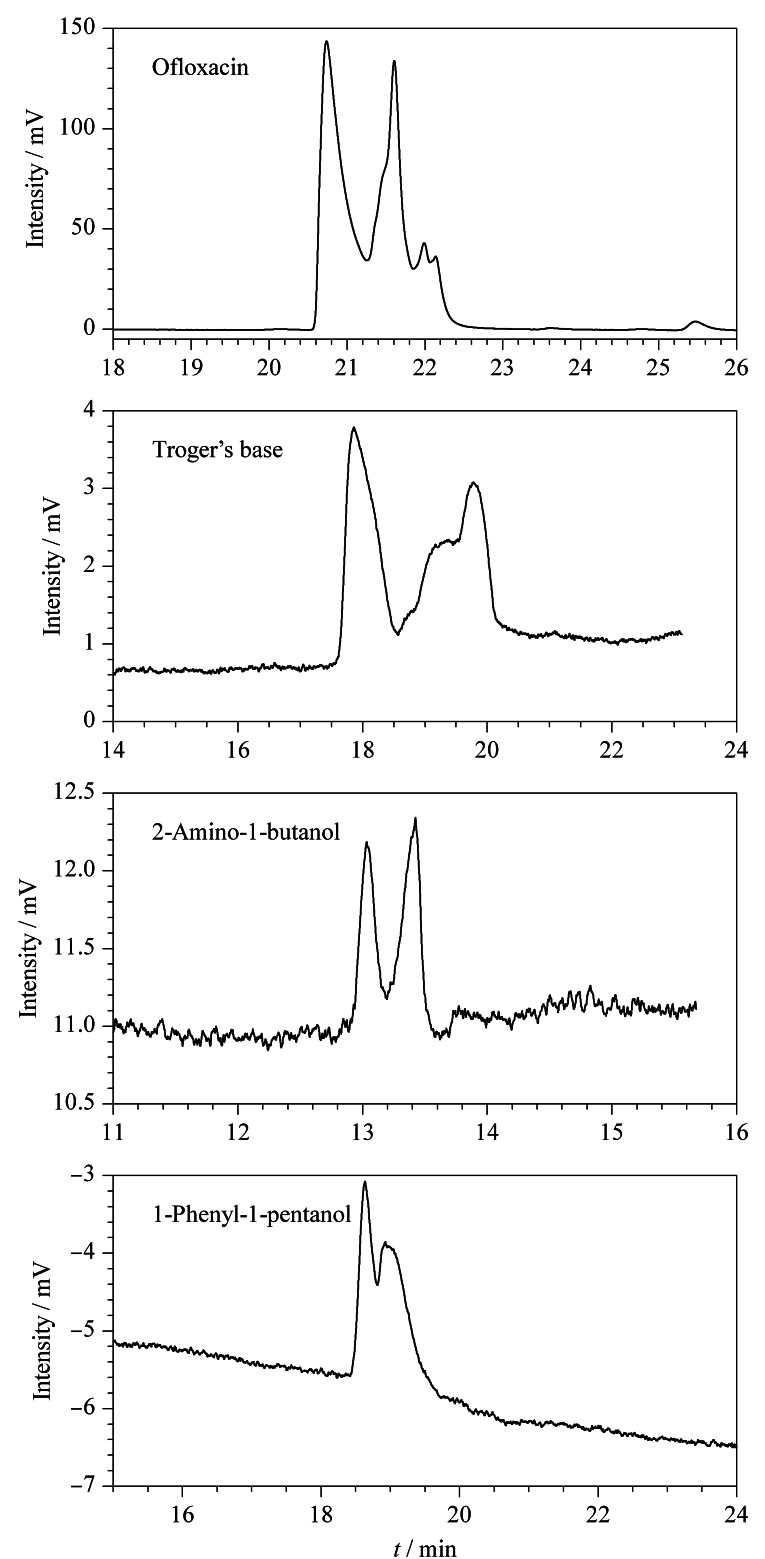
POCs手性柱对4种外消旋体的电色谱图

### 2.7 位置异构体在POCs电色谱柱上的拆分情况

POCs毛细管柱对位置异构体*o*,*m*,*p*-甲苯胺、*o*,*m*,*p*-氯苯胺也进行了分离研究,分离谱图见图9。电色谱分离条件为0.100 mol/L、pH=7.5的Tris-H_3_PO_4_缓冲溶液,分离电压为15 kV。位置异构体与POCs固定相间可能存在*π-π*键、偶极-偶极作用、立体构型匹配作用和氢键等相互作用力,从而使位置异构体分离。因位置异构体的分子结构、大小各不相同,故与固定相接触时产生的效果存在差异。

**图 9:**
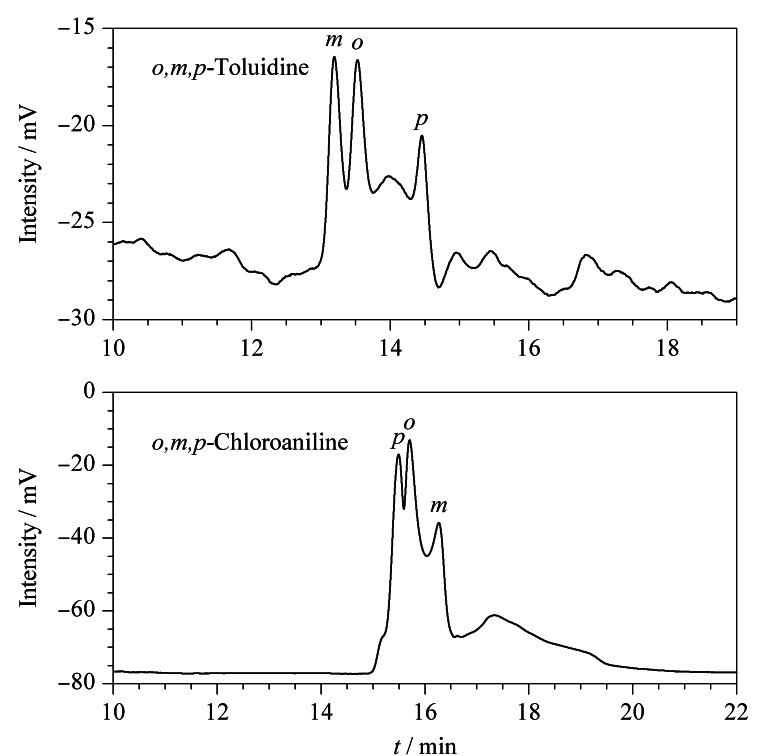
POCs手性柱对*o*,*m*,*p*-甲苯胺和*o*,*m*,*p*-氯苯胺的分离电色谱图

## 3 结论

将合成的多孔有机笼材料POCs涂覆在毛细管柱上制成相应的毛细管色谱手性柱。用该手性柱成功拆分了位置异构体*o*,*m*,*p*-氯苯胺和*o*,*m*,*p*-甲苯胺以及氧氟沙星、特罗格尔碱、2-氨基-1-丁醇和1-苯基-1-戊醇4种手性药物。实验表明,POCs作为CEC的手性固定相可行且具有一定的实用性,在拆分手性药物方面具有较大潜力。

## References

[1] Yuan L M. Chiral Recognition Material. 2nd ed. Beijing: Science Press, 2020

[2] Xie S M, Zhang Z J, Wang Z Y, et al. J Am Chen Soc, 2011, 133(31): 11892 10.1021/ja2044453 21751804

[3] Zhang H Z, Li F, Kang J W. Chinese Journal of Chromatography, 2023, 41(2): 160 36725712 10.3724/SP.J.1123.2022.07015 PMC9892973

[4] Liu M X, Li X J, Bai Y, et al. Chinese Journal of Chromatography, 2020, 38(3): 317 34213211 10.3724/SP.J.1123.2019.10019

[5] Hasell T, Chong S Y, Jelfs K E, et al. J Am Chem Soc, 2012, 134(1): 588 22080843 10.1021/ja209156v

[6] Lucero J M, Carreon M A. ACS Appl Mater, 2020, 12(28): 32182 10.1021/acsami.0c08040 32568506

[7] Barbour L J. Chem Commun, 2006, 11: 1163 10.1039/b515612m 16518481

[8] Jelfs K E, Wu X, Schmidtmann M, et al. Angew Chem Int Ed, 2011, 123(45): 10841 10.1002/anie.201105104 21928449

[9] Bojdys M J, Briggs M E, Jones J T A, et al. J Am Chem Soc, 2011, 133: 16566 21899280 10.1021/ja2056374

[10] Tozawa T, Jones J T A, Swamy S I, et al. Nat Mater, 2009, 8(12): 973 19855385 10.1038/nmat2545

[11] Jones J T A, Hasell T, Wu X, et al. Nature, 2011, 474: 367 21677756 10.1038/nature10125

[12] Song Q, Jiang S, Hasell T, et al. Adv Mater, 2016, 28(13): 2652 10.1002/adma.201505688 26800019

[13] Skowronek P, Gawronski J. Org Lett, 2008, 10: 4755 18837551 10.1021/ol801702j

[14] Zhang J H, Zhang M, Yuan L M, et al. Anal Methods, 2015, 7(8): 3448

[15] Kewley A, Stephenson A, Chen L, et al. Chem Mater, 2015, 27: 3207

[16] Tang M H. [MS Dissertation]. Kunming: Yunnan Normal University, 2021

[17] Zhang J H, Xie S M, Yuan L M, et al. Anal Chim Acta, 2018, 999: 169 29254569 10.1016/j.aca.2017.11.021

[18] Zhang L, Liang R, Hang C, et al. Green Chem, 2020, 22(8): 2498

[19] Jia W Y, Tang M H, Zhang J H, et al. Chinese Journal of Chromatography, 2022, 40(4): 391 35362687 10.3724/SP.J.1123.2021.07004 PMC9404018

